# Soaring migrants flexibly respond to sea-breeze in a migratory bottleneck: using first derivatives to identify behavioural adjustments over time

**DOI:** 10.1186/s40462-023-00402-4

**Published:** 2023-07-27

**Authors:** Paolo Becciu, David Troupin, Leonid Dinevich, Yossi Leshem, Nir Sapir

**Affiliations:** 1 Animal Flight Laboratory, Department of Evolutionary and Environmental Biology and Institute of Evolution, University of Haifa, 199 Aba Khoushy Ave. Mount Carmel, 3498838 Haifa, Israel; 2 Department of Ecology and Evolution, University of Lausanne, Lausanne, Switzerland; 3 Department of Zoology, George S. Wise Faculty of Life Sciences, University of Tel Aviv, 69978 Ramat Aviv, Tel Aviv, Israel

**Keywords:** Aeroecology, Behavioural optimization, Bird migration, Generalised additive models, Radar ornithology, Sea-breeze circulation, Weather radar, Wind drift

## Abstract

**Background:**

Millions of birds travel every year between Europe and Africa detouring ecological barriers and funnelling through migratory corridors where they face variable weather conditions. Little is known regarding the response of migrating birds to mesoscale meteorological processes during flight. Specifically, sea-breeze has a daily cycle that may directly influence the flight of diurnal migrants.

**Methods:**

We collected radar tracks of soaring migrants using modified weather radar in Latrun, central Israel, in 7 autumns between 2005 and 2016. We investigated how migrating soaring birds adjusted their flight speed and direction under the effects of daily sea-breeze circulation. We analysed the effects of wind on bird groundspeed, airspeed and the lateral component of the airspeed as a function of time of day using Generalized Additive Mixed Models. To identify when birds adjusted their response to the wind over time, we estimated first derivatives.

**Results:**

Using data collected during a total of 148 days, we characterised the diel dynamics of horizontal wind flow relative to the migration goal, finding a consistent rotational movement of the wind blowing towards the East (morning) and to the South-East (late afternoon), with highest crosswind speed around mid-day and increasing tailwinds towards late afternoon. Airspeed of radar detected birds decreased consistently with increasing tailwind and decreasing crosswinds from early afternoon, resulting in rather stable groundspeed of 16–17 m/s. In addition, birds fully compensated for lateral drift when crosswinds were at their maximum and slightly drifted with the wind when crosswinds decreased and tailwinds became more intense.

**Conclusions:**

Using a simple and broadly applicable statistical method, we studied how wind influences bird flight through speed adjustments over time, providing new insights regarding the flexible behavioural responses of soaring birds to wind conditions. These adjustments allowed the birds to compensate for lateral drift under crosswind and reduced their airspeed under tailwind. Our work enhances our understanding of how migrating birds respond to changing wind conditions during their long-distance journeys through migratory corridors.

**Supplementary Information:**

The online version contains supplementary material available at 10.1186/s40462-023-00402-4.

## Introduction

Geographic features and atmospheric conditions influence the movement of flying animals at different spatiotemporal scales [1, 2], yet little is known regarding the effects of mesoscale meteorological process on aerial flyers. Migrating birds, as well as other flying animals, may adjust their flight direction and speed in relation to weather and topography they encounter in order to accomplish their journey while saving time and energy. Nevertheless, these adjustments are challenging to assess under dynamic wind conditions. Importantly, the birds’ successful arrival at breeding or wintering grounds depends on their capacity to make space- and time-sensitive decisions to minimize their energetic cost of travel and the total duration of migration [3, 4]. This can be achieved by avoiding hindering weather conditions (e.g., headwinds) and exploiting advantageous ones (e.g., tailwinds) encountered *en route*, which presumably induce fitness-related benefits [5, 6].

Soaring land migrants often funnel into so-called migratory bottlenecks and corridors, which are characterized by geographic features that allow them to minimize their cost of transport [7–9], compared to nearby areas [10]. When flying through a migratory corridor or bottleneck, birds might still face both favourable and unfavourable weather conditions. Notably, the migrants could be impacted by winds that may blow at various speeds and come from different directions compared to the birds’ intended migration direction [11, 12].

Adverse winds, including headwinds and crosswinds that may increase bird flight energetics, could slow soaring birds down or even terminate their flight [12, 13] with possible consequences impacting their fitness. Soaring migrants can avoid strong crosswind and headwind by preferentially migrating only when favourable tailwinds prevail and no crosswind is present [14, 15]. However, if tailwinds are infrequent, waiting for ideal conditions could result in substantial delay of the journey [13, 16]. Initiating flight under crosswinds may result in drifting away from the intended track, with potentially severe consequences [17]. In-flight migrants may drift sideways due to crosswinds, or they can try to counter the lateral drift due to crosswind by orienting towards the incoming wind, to compensate fully or partially for the effect of the crosswind [12, 15, 18, 19]. Sideways drift compensation may diminish groundspeed, rendering it a suboptimal strategy [19]. Conversely, drifting birds may maximize groundspeed at the cost of geographic displacement, which may result in decreased fitness [17, 19].

Soaring migrants were found to flexibly change their flight speed and direction in relation to different wind conditions at different stages of their migration journey [15]. Specifically, Honey buzzards (*Pernis apivorus*) that crossed North-western Africa in autumn overcompensated for westward winds to circumvent the Atlas Mountains from their eastern side and then drifted with south-westward winds while crossing the Sahara Desert [15]. Probably, these different responses to wind allowed them to expend less energy and maximize their migration speed. Yet, many studies exploring the effects of wind on migrating birds involve between-day analyses that cannot usually portray within-day behavioural adjustments to dynamic wind conditions. Thus, we still do not know if and in what ways soaring migrants flexibly adjust their flight properties (e.g., direction, airspeed, etc.) at different times of the day to sustain high migration speed and maintain their intended migration direction while facing dynamic wind patterns such as a sea breeze. In addition, studies of soaring birds usually involve either studying a rather small number of individuals over large spatial scales using bird-borne tracking devices [2, 19] or researching massive migration passage at smaller spatial scales (1–20 km) using radar systems [20, 21]. How thousands of migrants adjust their flight to weather conditions when flying through a migration corridor is much less known.

The migration of soaring birds is massive along the Mediterranean coast of the Levant region where millions of soaring migrants are funnelled between the sea and the desert twice a year in a migration corridor parallel to the coastline [22, 23]. Along the coastline, especially in autumn, a dynamic sea-breeze circulation process prevails throughout the course of the day with progressively increasing westerlies and a clockwise change in wind direction towards the late afternoon and evening hours [24]. This process is created by the land-sea thermal gradient, and it is affected by several additional factors such as the Coriolis force, large scale pressure gradients and friction (see [25] for a comprehensive review of this process). The sea-breeze front advances in a direction perpendicular to the coast towards land and generates uplift with its vertical circulation component [24, 25]. The sea-breeze front has been hypothesised to influence the displacement of individual and flocks of birds in relation to the coastline. In Central Israel, birds were observed to align to the sea-breeze front, supposedly using the uplift to soar and increase their ground speed through the exploitation of this meteorologically dynamic process [26, 27].

The present study aims to examine how soaring birds are affected by the daily sea-breeze circulation during autumn migration, as well as their displacement relative to the coastline, using modified weather radar dedicated to bird studies in Central Israel. Specifically, we tested the following predictions: (a) if the wind rotates approximately from blowing eastwards to south-eastwards with the progression of the day, we expect that birds will reduce their airspeed over time and concomitantly increase their groundspeed under the elevated tailwind conditions. Conversely, we predict that airspeed will increase, and groundspeed is expected to decrease under strong crosswind and headwinds [3, 4, 28–30]. (b) Wind drift compensation is predicted when winds are constant during the journey [31, 32], while changes in wind speed and direction are predicted to induce variable behaviour, such as compensation for crosswinds that may otherwise drift the birds towards the desert (east of the migration corridor) due to sea-breeze [12].

We aim to predict movement of diurnal migrants with respect to sea-breeze circulation in an area that is characterized by intense soaring bird migration. Our work could be used as a framework to predict bird movement under meso-scale dynamic meteorological processes. We highlight the dynamic effects of the wind on bird flight and how they may change over time. [33]

## Materials and methods

### Radar data

**Fig. 1:**
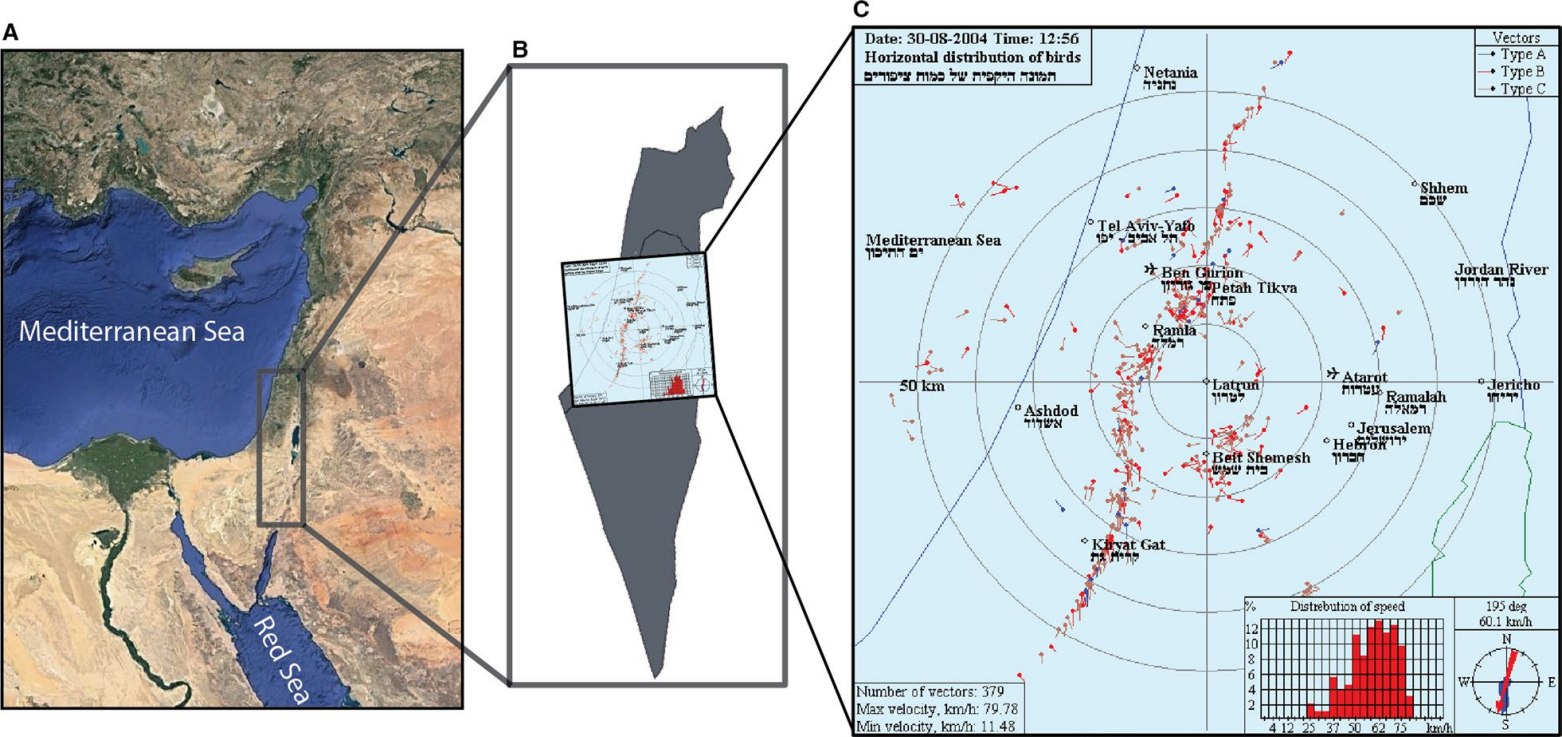
The study area located in the Levant region of the Middle East (**A**), where the radar is located in Central Israel (**B**), collecting bird tracking data between the Mediterranean Sea on the West, and the Jordan River and Dead Sea on the East (**C**). Mediterranean coast and the Jordan river are blue lines while the Dead Sea is delimited by a green line (panel **C**)

Data were gathered using a meteorological radar (MRL5) located in central Israel at Latrun (34.978° N, 31.839° E; Fig. 1), 18 km southeast of the Ben Gurion International Airport (Fig. 1C). The MRL5 meteorological radar operates at two different wavelengths (3 and 10 cm) using two high-grade transmitters and an antenna with two symmetrical narrow beams. The radius of the radar coverage area is 60 km (Fig. 1C). The narrow beam (0.5° on the 3 cm wavelength and 1.5° on the 10 cm wavelength) enables to determine the target coordinates and altitudes. For instance, at the distance of 50 km the automatic measurement accuracy on the 3.2 cm wavelength was around ± 200 m and at the distance of 20 km the accuracy was approximately ± 90 m [33]. This radar is also equipped with two supplementary devices, a device for measuring echo signal fluctuations and a polarization device [34]. This radar is the first meteorological radar adapted to exclusively detect birds with a series of algorithms that were applied to filter out other signals, such as those resulting from meteorological phenomena (i.e., clouds, rain, etc.) and human-related infrastructure and transportation (i.e., aircrafts, buildings, ships, and ground vehicles) [35]. It furthermore classifies detected single birds or small flocks into two main categories while providing their three-dimensional position and turning angle: local birds (low altitude and large turning angles) and migratory birds (higher altitudes and small turning angles). For a more detailed and technical explanation of the radar used in this study, please see [33–36]. This radar system was previously used to describe mean and range of altitudes and directions of nocturnal and diurnal bird migration over Israel [33, 34].

We selected only radar data of diurnal autumn bird migration collected from 3 h after sunrise to 3 h before sunset and focused on the time window of Honey buzzard (*Pernis apivorus*) migration between August 16 and September 30 [22]. We chose the Honey buzzard because it is the most abundant soaring migrant passing in the study area during this period, with more than 300,000 individuals counted on average in the autumn [22]. Other species of soaring migrants that pass through the area during this time of the year include black kites (*Milvus migrans*), levant sparrowhawks (*Accipiter brevipes*), white storks (*Ciconia ciconia*) and great white pelicans (*Pelecanus onocrotalus*) [22, 37, 38]. Data were collected and analysed between 2005 and 2016 from a total of 148 days of radar operation. We included data from the following 7 years (and total number of days of data collection in each year): 2005 (29 days), 2006 (32 days), 2007 (32 days), 2009 (8 days), 2014 (17 days), 2015 (13 days) and 2016 (17 days) (Additional file 1: Fig. S1). The years 2008, 2010, 2011, 2012, and 2013 contained fragmented and scarce data that were not used. The radar operated several days per week and never on weekends.

The radar was able to detect simultaneous echoes of birds and followed them in a 1.5-min time window, collecting speed (m/s), altitude (m) and coordinates of the birds at the starting and ending points of the time window [36]. Every 15–30 min a*.csv* table was produced with the targets data along with a map image. The final dataset contained a total of 857,206 tracks of diurnal soaring migrants.

### Weather data and movement parameters

We annotated the tracks with wind speed and direction from the closest weather station (Bet Dagan – 22 km west of the radar) of the Israeli Meteorological Service using a 10-min interval database (available at: https://ims.data.gov.il). Wind data were collected at 10 m above the ground. Radar data were annotated with the closest wind data in time. We calculated tailwind and crosswind relative to mean bird direction (187.7°) [39].

We calculated several flight parameters to explore the effects of wind on bird movement during migration. We used ground speed (V_g_ distance covered in time in m/s) calculated for the trajectory between the initial and ending points recorded by the radar. We calculated airspeed (V_a_) in m/s following Safi et al. (2013) [40]. To determine the birds' lateral speed in m/s, we define sideways speed as the lateral component of airspeed (LcA) with the following formula: V_s_ = V_a_ · sin(θ_b_–θ_m_), where θ_b_ is the track angle in radians [21]. θ_m_ is the mean direction of migration expressed in radians (− π, π). Furthermore, we calculated vertical speed as V_g_ sin(θ_s_), where θ_s_ is the slope angle between the beginning and the end point of a track considering distance covered horizontally and vertically. In addition, we calculated the linear distance between the end of each track and the coastline.

### Data analysis

Bird track parameters, which include groundspeed, airspeed, LcA and the wind components, were averaged per recording session of the radar, every 15 min, except for 6.25% of the cases in which the time lag between consecutive sessions was 30 min. We clustered our 857,206 tracks into 1977 sessions in which all the tracking parameters were averaged. These sessions constituted the final dataset for the analysis, to avoid temporal correlation of tracks recorded when conditions were similar for birds that flew at the same time within the radar coverage area.

In order to characterize and describe the hourly wind conditions and bird directions during the study period we used circular statistics (package *circular* [41] in the R environment [42]) such as the Rayleigh test to investigate the directionality of wind and bird directions. Then, we fitted two GLMMs to model wind directions (in radians) and wind speed as a function of time to sunset (as quadratic term) in interaction with year as a grouping factor. We used a random slope structure with ordinal date as a random factor using the function *glmmTMB* from the package *glmmTMB* [43] in R [42].

Furthermore, we fitted generalised additive mixed-effects models (GAMM) to assess how a) N–S and W–E components of the wind varied in their diurnal cycles (from 10 to 3 h before sunset), if b) groundspeed, c) airspeed and d) LcA varied with the progression of the day following the daily wind pattern. Also, we used GAMM to check if the e) compensation-drift behaviour of the birds in relation to wind changed with the hour of the day. In addition, we analysed also vertical speed and distance to the coastline with the progression of the day, these analyses are reported in the Supplementary Materials. We used cubic regression penalized smoothing basis (k = 12) for the smooth term “hours before sunset” as a numeric continuous variable (for example a value of − 6.25 means six hours and 15 min before the sunset), an autocorrelation-moving average correlation structure (*corARMA*, *p* = 2) for the “ordinal date”, and then used “year” as random intercept. In order to run the GAMMs, we used the function *gamm* from the package *mgcv* [44] in R [42], with the “L-BFGS-B” non-linear optimization method for parameter estimation [45]. Furthermore, to identify the time of behavioural change, we calculated the first derivative *f`(x)*, highlighting significant periods of positive or negative change in the relationships [46]. To do that, we used the function *derivatives* in *gratia* package [47].

The first derivative is the estimate (β̂) at each segment of the non-linear regression curve, which is the instantaneous rate of change of the function that defines the line. When the first derivative differs from 0, there is a positive or negative response, evaluated as significant when the 95% simultaneous intervals (calculated at each step) are not overlapping with 0. We calculated the first derivative on a new set of predicted values (N = 1000, the number of points used for evaluating the derivative) from the GAMMs and calculated the simultaneous intervals using n_sim = 10,000 (the number of simulations used in computing the simultaneous intervals). As finite difference (eps) we set a value of 1e−07. We used simultaneous confidence intervals since they include information on model reliability. Hence, they are much more informative than pointwise confidence intervals to assess the goodness of a model that, subsequently, can be used to make inference or predictions [48, 49]. An example code to run and plot the GAMM and its first derivative can be found in GitHub at https://github.com/paolobecciu/GAMMfirstderiv/, as well as the processed data used for the analyses. Descriptive statistics reported in the text are mean ± standard deviation, unless specified otherwise.

## Results

### The daily pattern of the wind during the migration period

**Fig. 2:**
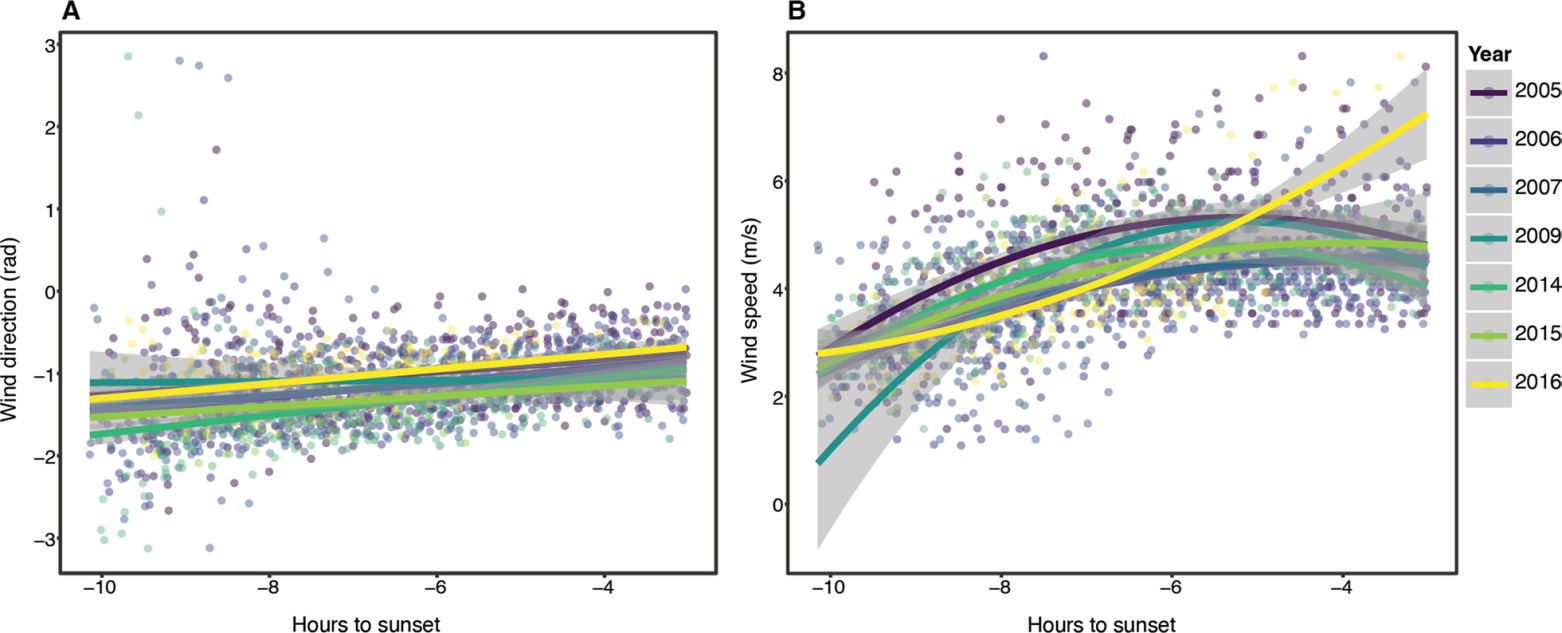
Wind direction (**A**) and speed (**B**) consistently change throughout the day with small variation between years. Regression slopes (with 95% C.I.) and colours highlight the different years of data collection

**Fig. 3:**
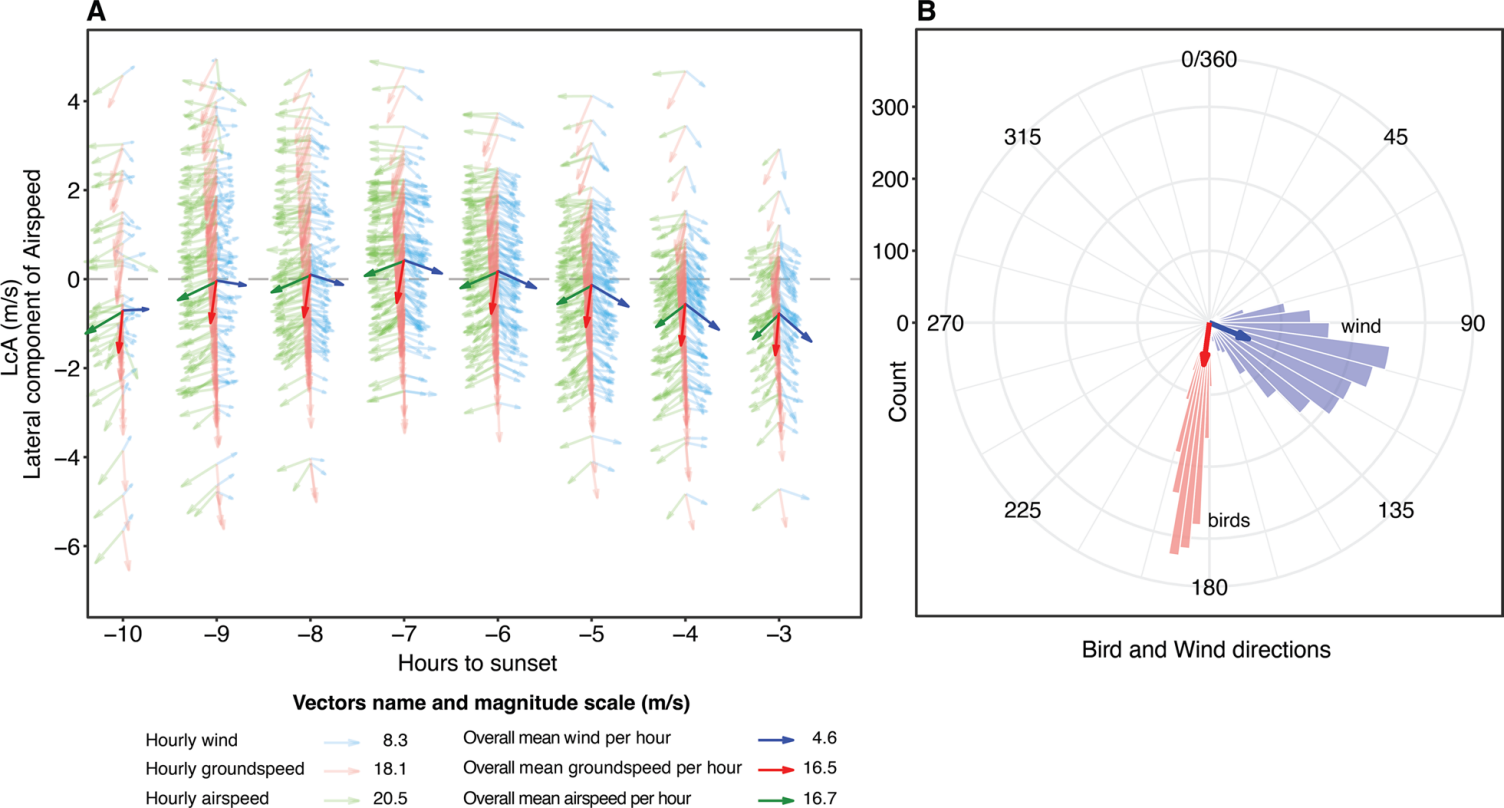
Visual representation of the flight directions and speeds of soaring migrants together with the wind conditions. Panel **A** shows the lateral component of airspeed (LcA) on the y-axis and its change throughout the day, with overlapped vectors of mean groundspeed (red), airspeed (green) and wind speed (blue). Note that partially transparent values indicate the variation for each hour including data from each day of tracking from all the 7 years used in this study (2005–07, 2009, 2014–16). For visual purposes the vectors are not in scale, see the legend to contextualize the vector magnitude. Dashed grey line with intercept y = 0 shows the full compensation line, where the groundspeed vectors align with the goal direction (187.7°). Panel **B** shows circular histograms of wind directions (blue) and soaring bird flight directions (red), the arrows represent the mean directions following Rayleigh tests (in which 0 = “uniform distribution” and 1 = “all the data are in the same direction”), which for the track birds is r = 0.99 (*p* < 0.0001) and for the winds is r = 0.90 (*p* < 0.0001)

Surface horizontal winds followed a consistent daily pattern during the study period showing a clockwise change of direction from the morning (about 10 h before sunset) blowing towards 87.1 ± 39.5 degrees, to the afternoon (about 3 h before sunset) blowing towards 131.1 ± 22.3 degrees. Mean wind direction was 112.5 ± 26.7 degrees (Rayleigh test: r = 0.898, *p* < 0.0001, Fig. 3B) with a mean wind speed of 4.1 ± 1.1 m/s. The results of the GLMMs regarding wind direction (in radians) and speed as function of time among the years, suggest that the wind direction changed slightly among years (see Additional file 1: Table S1 and S2). More importantly, the wind direction rotates during the day in the same way among the years (Fig. 2A). Furthermore, wind speed did not change among the years (notably, 2005 had a slightly higher average speed than the other years) and the increase in wind speed during the day followed a quadratic relationship with no between-year difference (except for 2016, see Fig. 2B).

**Fig. 4:**
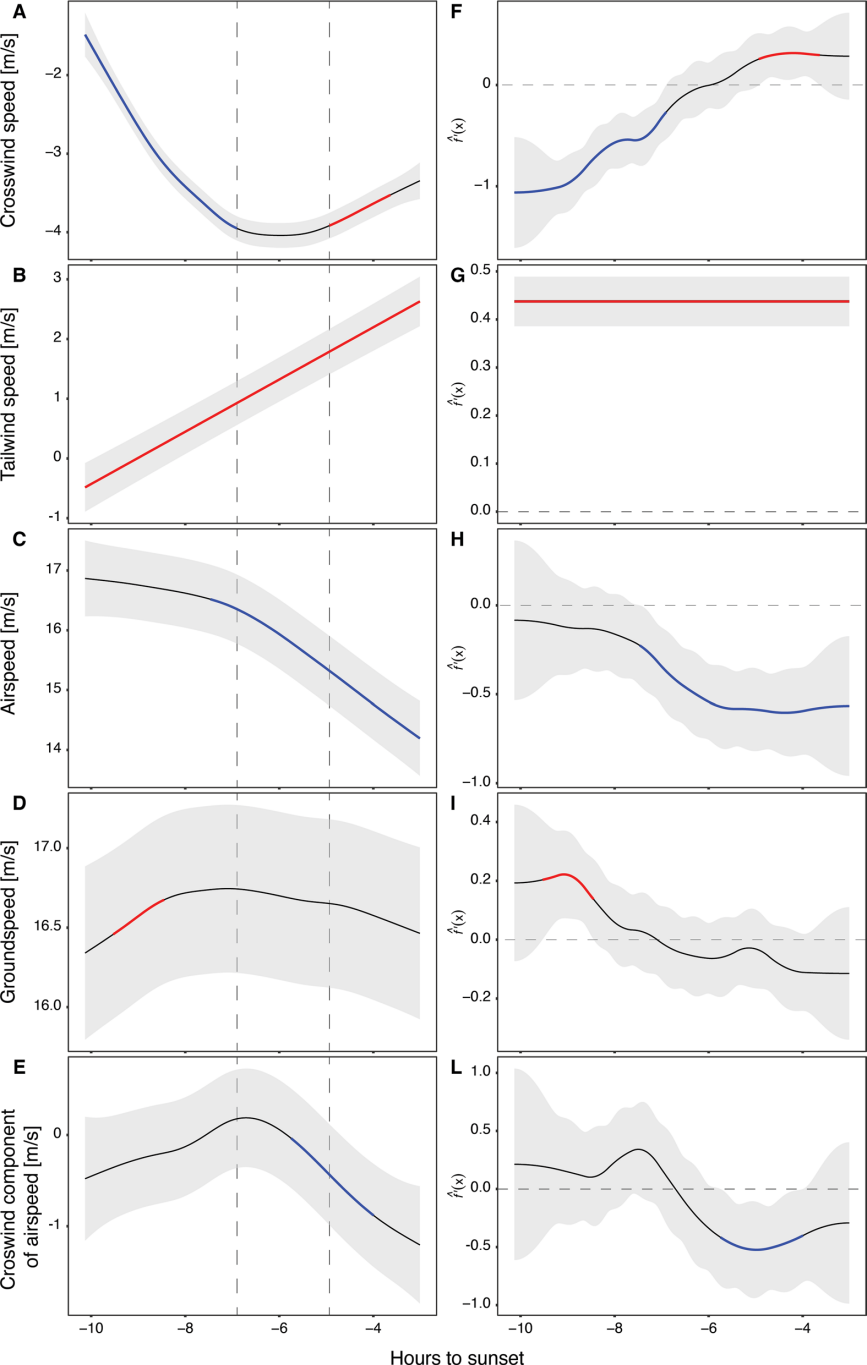
Cubic splines of the Generalized Additive Mixed Models with 95% C.I. (**A**–**E**) and their first derivative estimations and 95% simultaneous confidence intervals (**F**–**L**). Panels **A**–**E**: Estimated non-linear change of wind component and bird speeds (yaxis) as function of time (x-axis). Coloured sections indicate a significant rate of change that either increased (red) or decreased (blue) in that specific time period. The colouring of portions of the GAMM smoother line was adopted after calculating the first derivatives. The dashed lines delimitate the period of stronger crosswind component of the wind. Panels **F**–**L**: when the rate of change f’(x) in the y-axis is significantly above or below 0, the estimated rate of change is highlighted in blue (decrease) or red (increase)

The results of the GAMMs that examined the crosswind and tailwind components of the wind relative to the mean direction of the migrants (187.7°) as a function of hours before sunset, indicated that the daily wind patterns during the autumn was consistent among the 7 years included in the analysis. Crosswind speed increased from the beginning of the day (1 m/s), culminating in a plateau between the 7th and 5th hour before sunset (4 m/s), and then somewhat decreasing to 3.5 m/s towards the end of the day (Fig. 4A). The change in wind speed was the strongest before the peak (with a rate of change ranging between 0.2 and about 1 m/s per hour), while after the peak, crosswind speed decreased at a stable rate of 0.2–0.3 m/s per hour (Fig. 4F). Tailwind had a linear relationship (edf = 1.0; Additional file 1: Table S4) with the number of hours before sunset; hence this analysis was carried out as a linear model. Tailwind speed consistently increased throughout the day (Fig. 4B) at a stable rate of change of around 0.44 m/s per hour (Fig. 4G). These patterns demonstrate the shift in wind direction and speed throughout the day, with increasing wind speed and a shift between an air current flowing primarily from west to east reaching a peak in wind speed at the middle of the day and rotating southward, resulting in a stronger tailwind component at the later hours of the day (see Fig. 3).

### The daily pattern of movement of soaring migratory birds

Birds maintained a highly consistent daily track direction of 187.7 ± 0.1 degrees (Rayleigh test: r = 0.992, *p* < 0.0001) with a mean ground speed of 16.3 ± 0.8 m/s (Fig. 3). Generalized Additive Mixed Models (GAMMs) and calculated first derivatives allowed us to compare behavioural changes induced by daily wind condition patterns. We show that the predicted birds’ airspeed decreased during the day (Fig. 4C) essentially at two main rates of change (Fig. 4H): in the first hours of the day (− 10 to − 7) when tailwinds were weak (< 1 m/s) or even becoming headwinds (between − 1 and 0 m/s; Fig. 4B), the airspeed did not change from nearly 16.5–17 m/s. Then, with increasing crosswinds, airspeed started to decrease at a rate between 0.2 and 0.5 m/s (Fig. 4H), while when the crosswind peak has passed and tailwind increased, airspeed decreased at a rate of 0.5–0.6 m/s (Fig. 4G). Groundspeed was maintained between 16 and 17 m/s throughout the entire day (Fig. 4D), with a small change in the first two hours recorded (between − 10 and − 8; Fig. 4I). This change matched very low-speed winds and the birds’ highest airspeeds (Fig. 4A–C).

The birds’ LcA remained around zero with a non-significant peak around the time of crosswind speed peak, and then decreased towards the end of the day (Fig. 4E). Changes of LcA according to tailwind and crosswind components of the wind are reported in Additional file 1: Fig. S5. Vertical speed decreased in the first hours of the morning, from approximately 0.5–0 m/s, where it stayed stable for the rest of the day (Additional file 1: Fig. S3). Distance to the coast remained around 25–26 km, increasing significantly to 28–29 km at the end of the day (Additional file 1: Fig. S3).

## Discussion

With an unprecedented amount of more than 850,000 flight tracks from seven years, we quantitatively show how the flight of migrating soaring birds is predictable throughout the day and linked to a seasonal daily sea-breeze circulation pattern. We confirmed the characteristics of the diel pattern of the horizontal wind flow encountered by the birds during the autumn season [24], coinciding with the migration period of Honey buzzards (*Pernis apivorous*) over a key corridor located between two ecological barriers for migrating birds: the eastern basin of the Mediterranean Sea and the Syrian Desert [23]. The diel circulation of the wind, consistently blowing to the east at the beginning of the day and shifting towards south-east at the end of the day, with an increasing speed, seems to affect bird response. This includes a reduction in airspeed with increasing tailwinds, which resulted in a rather constant groundspeed. Furthermore, the lateral component of birds’ airspeed was around zero suggesting nearly full compensation when crosswind was the strongest, while the birds were partially drifting with the lateral airflow when crosswind started to slow down and tailwind increased. These flight responses to predictable weather conditions probably allow soaring birds to minimize their cost of transport (at least for most of the day) and keep a constant migration speed of around 16.5 m/s which would account for roughly 415 km covered per day, assuming an average of 7 h of active migration per day in this section of their migration flyway [50]. By fully compensating for or drifting with the wind, soaring birds kept the migration direction towards the south-south-west parallel to the coastline [15, 51], in a way similar to nocturnal migrating songbirds flying along the Eastern U.S. coastline [52]. Notably, we cannot fully assess if their strategy was optimal in terms of energy minimization, since we could not analyse an important component of this behaviour related to altitude selection [53].

Our results confirm our first prediction regarding airspeed adjustment. As the wind rotated throughout the day, birds exploited the resulting increasing tailwind vector by decreasing their airspeed at a similar rate. Since all the species of soaring migrants migrate roughly at the same hours of the day which are included in our analyses, we do not think this pattern reflects a change in the composition of migrating species during the day. Noteworthy, groundspeed did not increase with tailwinds but resulted in rather stable speed throughout the entire day. Hence, soaring migrants maintain a rather constant groundspeed under different winds. This may suggest that there is a cost for increased groundspeed—either a cost that relates to risks of grounding or switching to flapping flight that may bear metabolic expenses [20], or other possible costs associated with high groundspeed such as reduced flight control, hampered navigation and reduced sensing of subtle changes in airflow during flight, which might limit the use of convective updrafts for soaring [1, 20].

The soaring migrants’ lateral component of airspeed was mostly around zero when wind blew at different intensities towards the east (from sea to desert) in the first four hours of the day, although few radar sessions recording at high crosswind speeds showed events of drifting (see Additional file 1: Fig. S5). To keep the migration direction and avoid wind drift, the birds compensated with dynamic modulation of their LcA. At the highest mean crosswind value (4 m/s) airspeed was approximately 16 m/s and only 3% lower than groundspeed. In this situation, the combination of crosswind and tailwind components do not seem to induce major costs to maintain the preferred direction. In the following three hours, the clockwise turning of the wind offered decreasing crosswinds and increasing tailwinds that lowered the birds’ lateral component of airspeed, showing a partial compensation (airspeed/groundspeed ratio around 90%), but still within an angle around the mean migration direction (187.7°). Obviously, in cases of high tailwind speeds, when crosswinds are around zero, LcA is also around 0 (Additional file 1: Fig. S5). Overall, during this time birds were drifting eastward to a small extent, resulting in a mean difference distance from the coastline of only 3 km between the morning and the afternoon (see Additional file 1: Fig. S3). In addition, vertical speed could suggest a use of linear soaring (after a peak of positive vertical speed in the morning probably associated with the birds roosting in the area using the first thermals), which may be a result of good thermal conditions (Additional file 1: Fig. S3) [26].

Our results are neither confirming nor rejecting the interesting hypothesis of Alpert et al. [26] suggesting that soaring migrants could probably ride the updrafts generated by the sea-breeze front moving eastwards, and further research is needed in the future to confirm this idea. But importantly, we highlight that flight response to sea-breeze allowed birds to reduce drifting eastwards such that they would then need to cross a wide sea later during their journey, after being drifted to South Sinai or even the Arabian Peninsula, as opposed to crossing the Suez Canal in North Sinai over a continuous landmass. Interestingly, by migrating parallel to the coastline, the birds flew over the southern coastal plains of Israel and the northern part of Sinai, areas that contain trees, and thus seem suitable for safe roosting above ground before taking the leap over the Sahara Desert of which many areas are devoid of trees, making the chosen route preferable also in this respect [22, 27].

The method used in this study could help address current challenges posed by high-resolution animal movement data [54, 55], by helping to interpret behavioural change over time in relation to dynamic environmental conditions (see [18, 56]). Furthermore, this method is not constrained by any timescale as long as the periodicity of the variable is accounted for by the GAMM. It may consequently be used for long (millennia) as well as short (minutes and seconds) time series [46, 57, 58]. Furthermore, this method is easily applicable to highlight any changes over time per se, either a behavioural change or in relation to an event happening to the focal individual or population considered (e.g., predator–prey interaction, arrival to a specific location, inter- and intraspecific contacts, etc.) [56, 59].

This study was possible because of an exceptional adaptation of the MRL-5 weather radar, which was applied to detect only biological targets and filtered out weather and human-related features [33–36]. This allowed the collection of an enormous quantity of bird migration data. Diurnal bird migration includes over 850,000 tracks and many more nocturnal migration tracks were recorded by this system. The collected data isolates the target, locks on it for a certain amount of time and allows the creation of a short track. This output is comparable (albeit producing shorter tracks) to those of modified marine radars or tracking radars, with the advantage of scanning a 10- to 100-fold larger area. Therefore, the combination of having precise movement data of a huge number of targets for several years allowed us to clearly quantify behavioural within-day variation and explore how flying migrants respond to their environment in one of the busiest migration corridors in the entire globe [60].

Importantly, this dataset may address additional questions in the study of bird migration, providing extremely valuable information regarding the risk of bird strikes near several military and civil airports with heavy aircraft traffic. Our work provides important insights for understanding and predicting diurnal migration movements over an area where a significant number of collisions between aircrafts and birds take place every year (total of 402 bird strikes between 2000 and 2016), causing severe economic losses and risking human lives [61]. Knowing and predicting bird movement and response to wind conditions (i.e., ground-, sideways- and airspeed modulation relative to the wind) may additionally be helpful for predicting movement not only over central Israel but also over other areas of the Levant region and elsewhere in the world, where migrating birds face similar sea-breeze circulations [62].

## Supplementary Information

**Additional file 1**. Supporting figures and tables for Methods and Results.

## Data Availability

The data used for the analyses of this study are available at https://github.com/paolobecciu/GAMMfirstderiv/.
