# Supplementary material for: Soaring migrants flexibly respond to sea-breeze in a migratory bottleneck: using first derivatives to identify behavioural adjustments over time
Source: Mov Ecol. 2023 Jul 27;11:44. doi: 10.1186/s40462-023-00402-4 (PMC10375660; doi:10.1186/s40462-023-00402-4)
Supplement: Supplementary file 1 — Additional file 1. Supporting figures and tables for Methods and Results. [file 40462_2023_402_MOESM1_ESM.docx]

# Electronic Supplementary Materials

# Soaring migrants flexibly respond to sea-breeze in a migratory bottleneck: using first derivatives to identify behavioural adjustments over time

Paolo Becciu^1,2,*^, David Troupin^1^, Leonid Dinevich^3^, Yossi Leshem^3^, Nir Sapir^1^

^1^ Animal Flight Laboratory, Department of Evolutionary and Environmental Biology and Institute of Evolution, University of Haifa, 199 Aba Khoushy Ave. Mount Carmel, 3498838 Haifa, Israel.

^2^ Department of Ecology and Evolution, University of Lausanne, Lausanne, Switzerland.

^3^ George S. Wise Faculty of Life Sciences, Department of Zoology, University of Tel Aviv, 69978 Ramat Aviv, Tel Aviv, Israel.

^*^ [paolo.becciu@unil.ch](mailto:paolo.becciu@unil.ch); [pbecciu89@gmail.com](mailto:pbecciu89@gmail.com)

Content:

- **Fig. S1 –** Radar tracks count and operating radar timeline
- **Fig. S2 –** Descriptive plots with data used for the GAMMs
- **Tables S1-S2** **–** GLMM tables
- **Tables S3-S9** **–** GAMM tables
- **Fig. S3 –** GAMM plots and first derivatives of 2 parameters not reported in the main text: “vertical speed” and “distance to the coast”
- **Fig.** **S4** – Circular plots per hour of bird and wind directions
- **Fig**. **S5** – Relationship between LcA, crosswinds and tailwinds


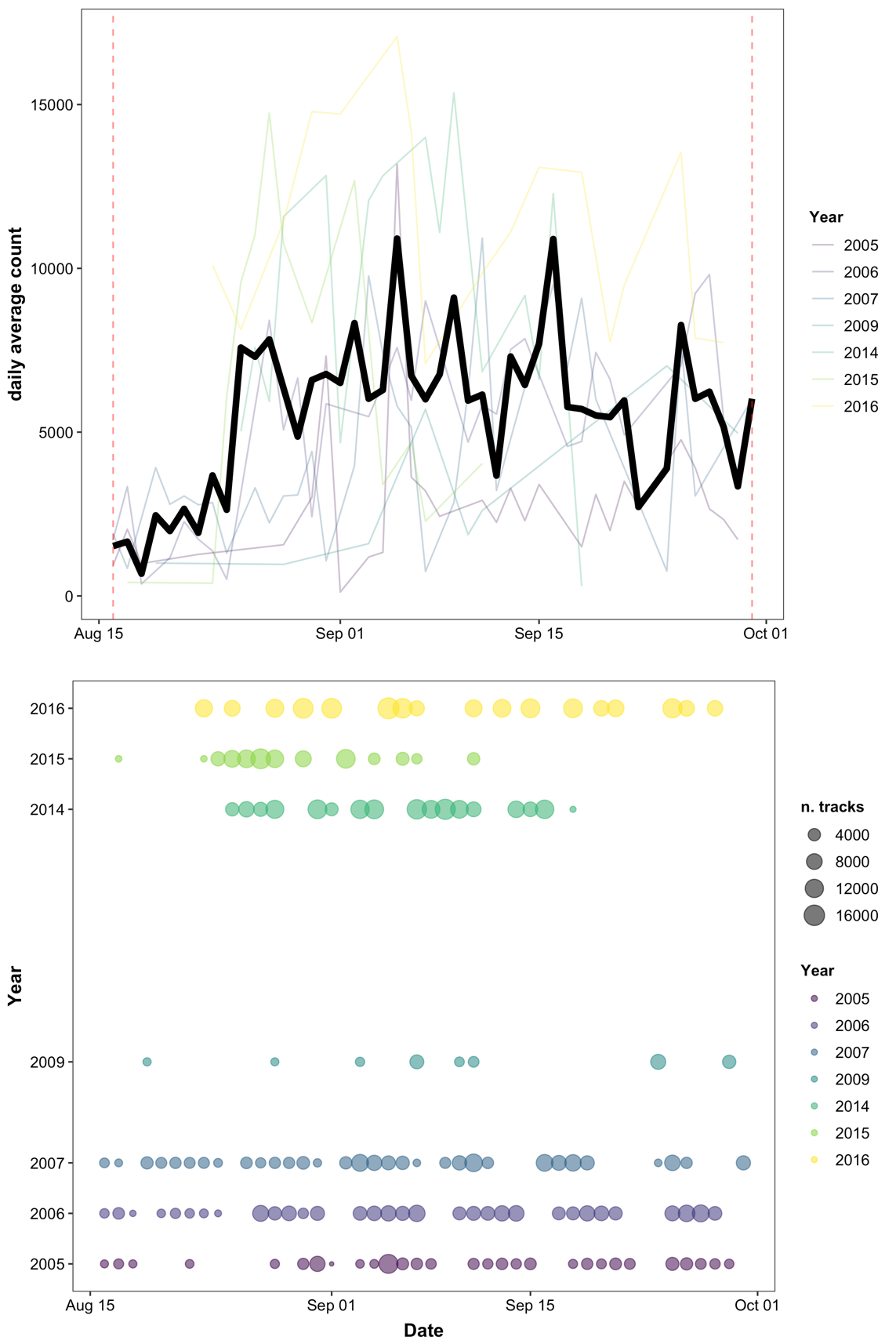


Fig. S1 – Top panel: Count of recorded radar tracks over the years included in this study. The thicker black line is the average count of radar tracks per day in the years of the study (2005-07, 2009, 2014-16). The dashed lines delimit the time window considered for the Honey Buzzard migration (15 Aug - 30 Sep). Bottom panel: Operating days of the radar in the study period. Each circle corresponds to a day of the year, and the size is the sum of radar tracks recorded that day.


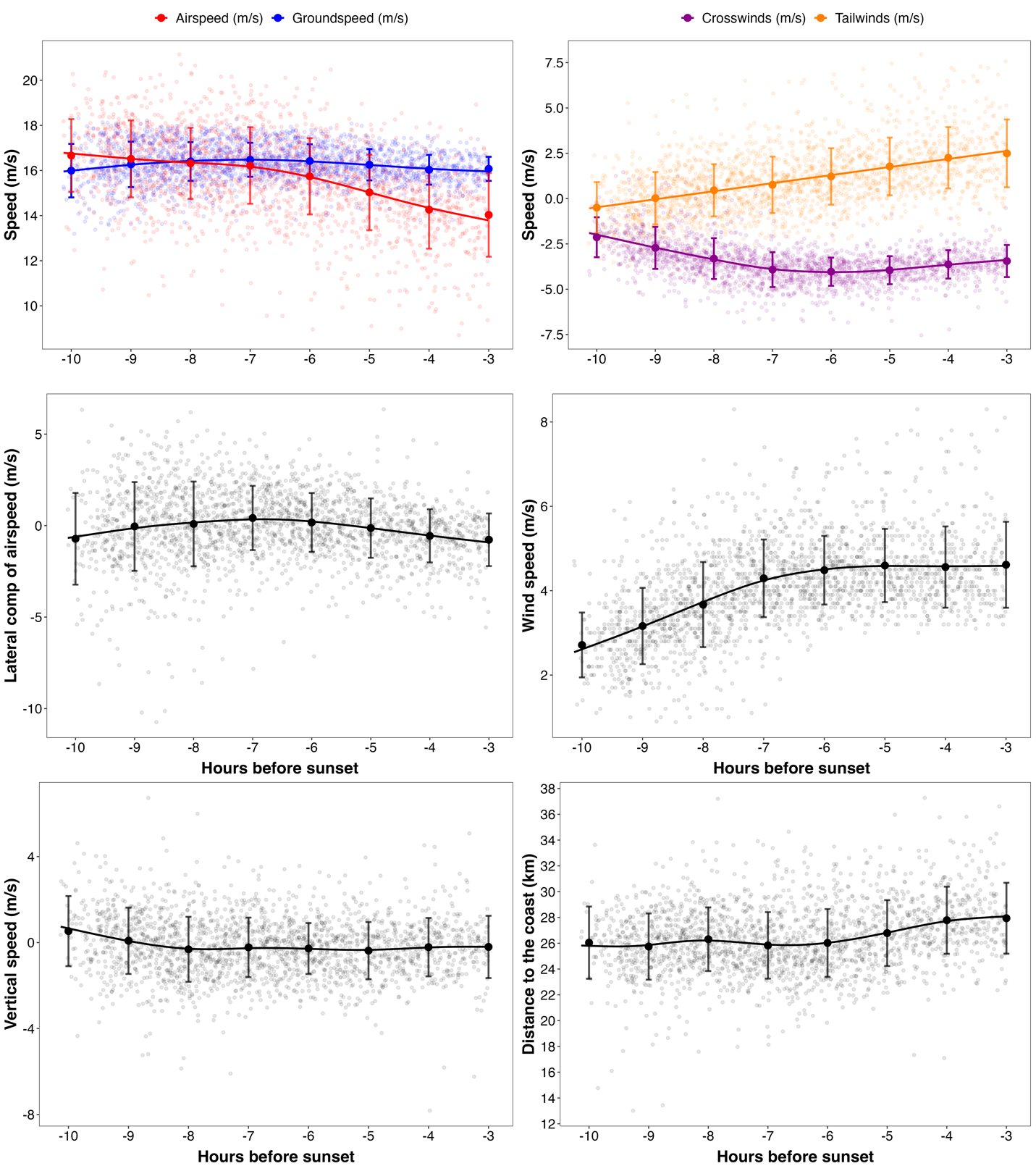


Fig. S2 – Graphs illustrating the data used from each radar recording session (every 15-30 minutes, n = 1977 data points). Bigger dots are mean per hour with error bars showing standard deviation and a gam smoother (y ~ s(x)) highlighting the trend of the data.

## Model tables

**Wind direction GLMM table**

| **group** |  | **Estimate** | **Standard Error** |  | **statistic** | **p-value** |  |
| --- | --- | --- | --- | --- | --- | --- | --- |
| Fixed effects | | | | | | | |
|  | (Intercept) | -1.055 | 0.038 |  | -27.821 | 0.0000 | *** |
|  | scale(h.from.sunset) | 0.145 | 0.019 |  | 7.712 | 0.0000 | *** |
|  | yearf2006 | -0.141 | 0.030 |  | -4.643 | 0.0000 | *** |
|  | yearf2007 | -0.102 | 0.033 |  | -3.121 | 0.0018 | ** |
|  | yearf2009 | -0.148 | 0.081 |  | -1.823 | 0.0683 | . |
|  | yearf2014 | -0.242 | 0.038 |  | -6.320 | 0.0000 | *** |
|  | yearf2015 | -0.064 | 0.057 |  | -1.108 | 0.2677 |  |
|  | yearf2016 | 0.073 | 0.041 |  | 1.759 | 0.0785 | . |
| Random effects | | | | | | | |
| n.days | sd__(Intercept) | 0.195 |  |  |  |  |  |
| n.days | sd__scale(h.from.sunset) | 0.105 |  |  |  |  |  |
| n.days | cor__(Intercept).scale(h.from.sunset) | -0.667 |  |  |  |  |  |
| Residual | sd__Observation | 0.420 |  |  |  |  |  |
| R^2^ (marginal) |  | 0.11 |  |  |  |  |  |
| R^2^ (conditional) |  | 0.30 |  |  |  |  |  |
| Signif. codes: 0 <= '***' < 0.001 < '**' < 0.01 < '*' < 0.05 | | | | | | | |
|  | | | | | | | |

Tab. S1 – Summary table of the GLMM with wind direction in radians as a response variable. The table reports the relationship between time of the day (hours before sunset), year and the changing wind direction (see Fig. 2A in main text). Julian date was included as random factor.

**Wind speed GLMM table**

| **group** |  | **Estimate** | **Standard Error** |  | **statistic** | **p-value** |  |
| --- | --- | --- | --- | --- | --- | --- | --- |
| Fixed effects | | | | | | | |
|  | (Intercept) | 4.635 | 0.078 |  | 59.686 | 0.0000 | *** |
|  | scale(h.from.sunset) | -2.527 | 0.301 |  | -8.386 | 0.0000 | *** |
|  | yearf2006 | -0.804 | 0.054 |  | -14.821 | 0.0000 | *** |
|  | yearf2007 | -0.743 | 0.058 |  | -12.725 | 0.0000 | *** |
|  | yearf2009 | -0.736 | 0.167 |  | -4.417 | 0.0000 | *** |
|  | yearf2014 | -0.703 | 0.068 |  | -10.282 | 0.0000 | *** |
|  | yearf2015 | -0.805 | 0.127 |  | -6.317 | 0.0000 | *** |
|  | yearf2016 | -0.315 | 0.089 |  | -3.545 | 0.0004 | *** |
|  | I(scale(h.from.sunset^2)) | -3.039 | 0.306 |  | -9.934 | 0.0000 | *** |
|  | scale(h.from.sunset):yearf2006 | 1.135 | 0.371 |  | 3.055 | 0.0022 | ** |
|  | scale(h.from.sunset):yearf2007 | 1.464 | 0.383 |  | 3.823 | 0.0001 | *** |
|  | scale(h.from.sunset):yearf2009 | -0.987 | 1.119 |  | -0.882 | 0.3776 |  |
|  | scale(h.from.sunset):yearf2014 | -0.263 | 0.473 |  | -0.556 | 0.5780 |  |
|  | scale(h.from.sunset):yearf2015 | 1.117 | 0.971 |  | 1.151 | 0.2499 |  |
|  | scale(h.from.sunset):yearf2016 | 4.813 | 0.743 |  | 6.475 | 0.0000 | *** |
|  | yearf2006:I(scale(h.from.sunset^2)) | 1.097 | 0.381 |  | 2.880 | 0.0040 | ** |
|  | yearf2007:I(scale(h.from.sunset^2)) | 1.410 | 0.386 |  | 3.655 | 0.0003 | *** |
|  | yearf2009:I(scale(h.from.sunset^2)) | -1.342 | 1.236 |  | -1.086 | 0.2777 |  |
|  | yearf2014:I(scale(h.from.sunset^2)) | -0.247 | 0.472 |  | -0.525 | 0.5997 |  |
|  | yearf2015:I(scale(h.from.sunset^2)) | 0.976 | 0.874 |  | 1.117 | 0.2639 |  |
|  | yearf2016:I(scale(h.from.sunset^2)) | 4.147 | 0.693 |  | 5.982 | 0.0000 | *** |
| Random effects | | | | | | | |
| n.days | sd__(Intercept) | 0.431 |  |  |  |  |  |
| n.days | sd__scale(h.from.sunset) | 0.293 |  |  |  |  |  |
| n.days | cor__(Intercept).scale(h.from.sunset) | -0.423 |  |  |  |  |  |
| Residual | sd__Observation | 0.736 |  |  |  |  |  |
| R^2^ (marginal) |  | 0.39 |  |  |  |  |  |
| R^2^ (conditional) |  | 0.60 |  |  |  |  |  |
| Signif. codes: 0 <= '***' < 0.001 < '**' < 0.01 < '*' < 0.05 | | | | | | | |
|  | | | | | | | |

Tab. S2 – Summary table of the GLMM with wind speed (m/s) as a response variable. The table reports the relationship between time of the day (hours before sunset) and its quadratic term in interaction with the year and the changing wind speed (see Fig. 2B in main text). Julian date was included as random factor.

**Crosswinds GAMM table**

| **Component** | **Term** | **Estimate** | **Std Error** | **t-value** | **p-value** |  |
| --- | --- | --- | --- | --- | --- | --- |
| A. parametric coefficients | (Intercept) | -3.516 | 0.060 | -58.842 | 0.0000 | *** |
| **Component** | **Term** | **edf** | **Ref. df** | **F-value** | **p-value** |  |
| B. smooth terms | s(h.from.sunset) | 6.712 | 6.712 | 61.033 | 0.0000 | *** |
| Signif. codes: 0 <= '***' < 0.001 < '**' < 0.01 < '*' < 0.05 | | | | | | |
|  | | | | | | |
| Adjusted R-squared: 0.23 | | | | | | |
| N: 1977 | | | | | | |

Tab. S3 – Summary table of the GAMM with crosswind component of the wind as a response variable. The table reports the intercept and the complexity of the fitted cubic spline (edf).

**Tailwinds GAMM table**

| **Component** | **Term** | **Estimate** | **Std Error** | **t-value** | **p-value** |  |
| --- | --- | --- | --- | --- | --- | --- |
| A. parametric coefficients | (Intercept) | 3.947 | 0.257 | 15.349 | 0.0000 | *** |
|  | h.from.sunset | 0.437 | 0.026 | 16.696 | 0.0000 | *** |
| Signif. codes: 0 <= '***' < 0.001 < '**' < 0.01 < '*' < 0.05 | | | | | | |
|  | | | | | | |
| Adjusted R-squared: 0.22 | | | | | | |
| N: 1977 | | | | | | |

Tab. S4 – Summary table of the GAMM with tailwind component of the wind as a response variable. In this case the GAMM is containing only a parametric coefficient, hence it is equal to a Linear Mixed Model.

**Airspeed GAMM table**

| **Component** | **Term** | **Estimate** | **Std Error** | **t-value** | **p-value** |  |
| --- | --- | --- | --- | --- | --- | --- |
| A. parametric coefficients | (Intercept) | 16.004 | 0.288 | 55.535 | 0.0000 | *** |
| **Component** | **Term** | **edf** | **Ref. df** | **F-value** | **p-value** |  |
| B. smooth terms | s(h.from.sunset) | 4.205 | 4.205 | 50.011 | 0.0000 | *** |
| Signif. codes: 0 <= '***' < 0.001 < '**' < 0.01 < '*' < 0.05 | | | | | | |
|  | | | | | | |
| Adjusted R-squared: 0.19 | | | | | | |
| N: 1977 | | | | | | |

Tab. S5 – Summary table of the GAMM with bird airspeed as a response variable. The table reports the intercept and the complexity of the fitted cubic spline (edf).

**Groundspeed GAMM table**

| **Component** | **Term** | **Estimate** | **Std Error** | **t-value** | **p-value** |  |
| --- | --- | --- | --- | --- | --- | --- |
| A. parametric coefficients | (Intercept) | 16.648 | 0.267 | 62.245 | < 0.0001 | *** |
| **Component** | **Term** | **edf** | **Ref. df** | **F-value** | **p-value** |  |
| B. smooth terms | s(h.from.sunset) | 4.940 | 4.940 | 6.771 | < 0.0001 | *** |
| Signif. codes: 0 <= '***' < 0.001 < '**' < 0.01 < '*' < 0.05 | | | | | | |
|  | | | | | | |
| Adjusted R-squared: 0.025 | | | | | | |
| N: 1977 | | | | | | |

Tab. S6 – Summary table of the GAMM with bird groundspeed as a response variable. The table reports the intercept and the complexity of the fitted cubic spline (edf).

**LcA – Lateral component of Airspeed GAMM table**

| **Component** | **Term** | **Estimate** | **Std Error** | **t-value** | **p-value** |  |
| --- | --- | --- | --- | --- | --- | --- |
| A. parametric coefficients | (Intercept) | -0.253 | 0.259 | -0.976 | 0.3294 |  |
| **Component** | **Term** | **edf** | **Ref. df** | **F-value** | **p-value** |  |
| B. smooth terms | s(h.from.sunset) | 5.068 | 5.068 | 8.434 | 0.0000 | *** |
| Signif. codes: 0 <= '***' < 0.001 < '**' < 0.01 < '*' < 0.05 | | | | | | |
|  | | | | | | |
| Adjusted R-squared: 0.03 | | | | | | |
| N: 1977 | | | | | | |

Tab. S7 – Summary table of the GAMM with bird lateral component of airspeed (LcA) as a response variable. The table reports the intercept and the complexity of the fitted cubic spline (edf).

**Vertical speed GAMM table**

| **Component** | **Term** | **Estimate** | **Std Error** | **t-value** | **p-value** |  |
| --- | --- | --- | --- | --- | --- | --- |
| A. parametric coefficients | (Intercept) | -0.139 | 0.164 | -0.849 | 0.3959 |  |
| **Component** | **Term** | **edf** | **Ref. df** | **F-value** | **p-value** |  |
| B. smooth terms | s(h.from.sunset) | 4.415 | 4.415 | 4.638 | 0.0005 | *** |
| Signif. codes: 0 <= '***' < 0.001 < '**' < 0.01 < '*' < 0.05 | | | | | | |
|  | | | | | | |
| Adjusted R-squared: 0.02 | | | | | | |
| N: 1977 | | | | | | |

Tab. S8 – Summary table of the GAMM with bird vertical speed as a response variable. The table reports the intercept and the complexity of the fitted cubic spline (edf).

**Distance to the coast GAMM table**

| **Component** | **Term** | **Estimate** | **Std Error** | **t-value** | **p-value** |  |
| --- | --- | --- | --- | --- | --- | --- |
| A. parametric coefficients | (Intercept) | 26.333 | 0.276 | 95.366 | 0.0000 | *** |
| **Component** | **Term** | **edf** | **Ref. df** | **F-value** | **p-value** |  |
| B. smooth terms | s(h.from.sunset) | 6.771 | 6.771 | 14.271 | 0.0000 | *** |
| Signif. codes: 0 <= '***' < 0.001 < '**' < 0.01 < '*' < 0.05 | | | | | | |
|  | | | | | | |
| Adjusted R-squared: 0.06 | | | | | | |
| N: 1977 | | | | | | |

Tab. S9 – Summary table of the GAMM with bird distance to the coast as a response variable. The table reports the intercept and the complexity of the fitted cubic spline (edf).

## Panel figure – Vertical speed and Distance to the coast GAMMS and first derivatives


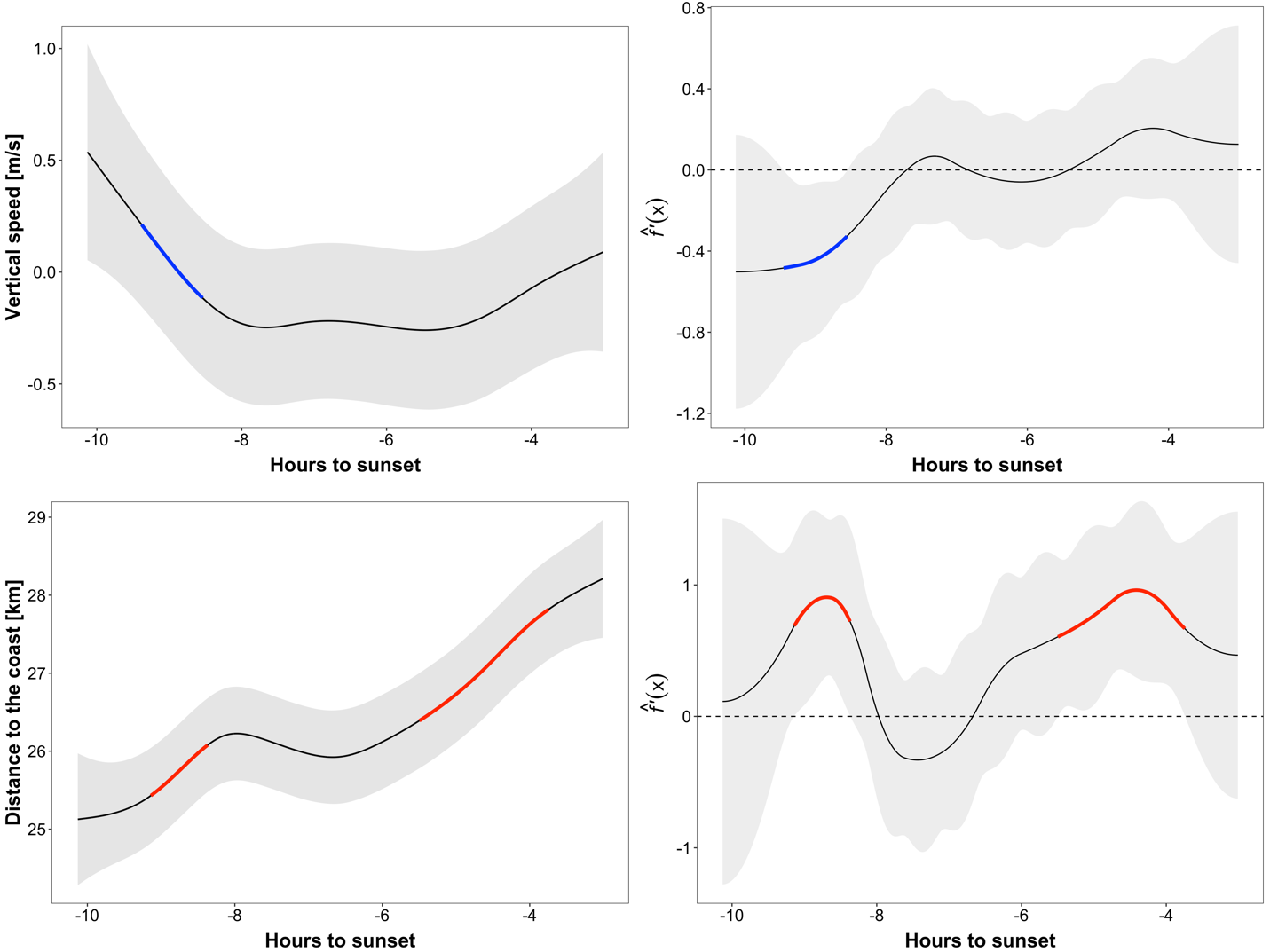


Fig. S3 – Cubic splines of the Generalized Additive Mixed Models with 95% C.I. (left side panels) and their first derivative estimations and 95% simultaneous confidence intervals (right side panels). Left panels: Estimated non-linear change of vertical speed and distance to the coast (y-axis) as function of time (x-axis), coloured portions depend on the rate of change that significantly increased (red) or decreased (blue) in that specific time period. The colouring of portions of the GAMM smoother line was adopted after calculating the first derivatives. Right panels: when the rate of change f’(x) in the y-axis is above or below 0 (including the 95% C.I.) the estimated rate of change is highlighted in blue (decrease) or red (increase).

## Panel figure – Circular plots per hour of bird and wind directions


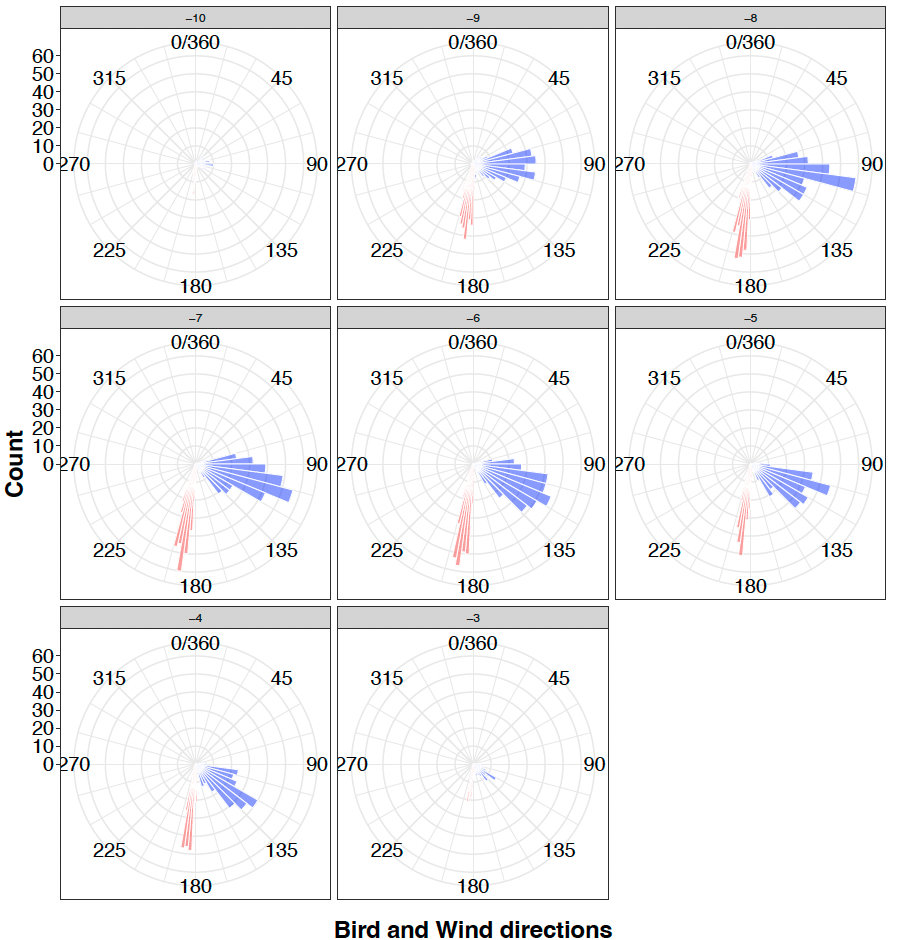


Fig. S4 – Circular histograms of wind directions (blue) and soaring bird flight directions (red) per hour (hours before sunset expressed in negative values, 0 being the sunset). See Fig. 3B in the main text for an overall summary of wind and bird directions.


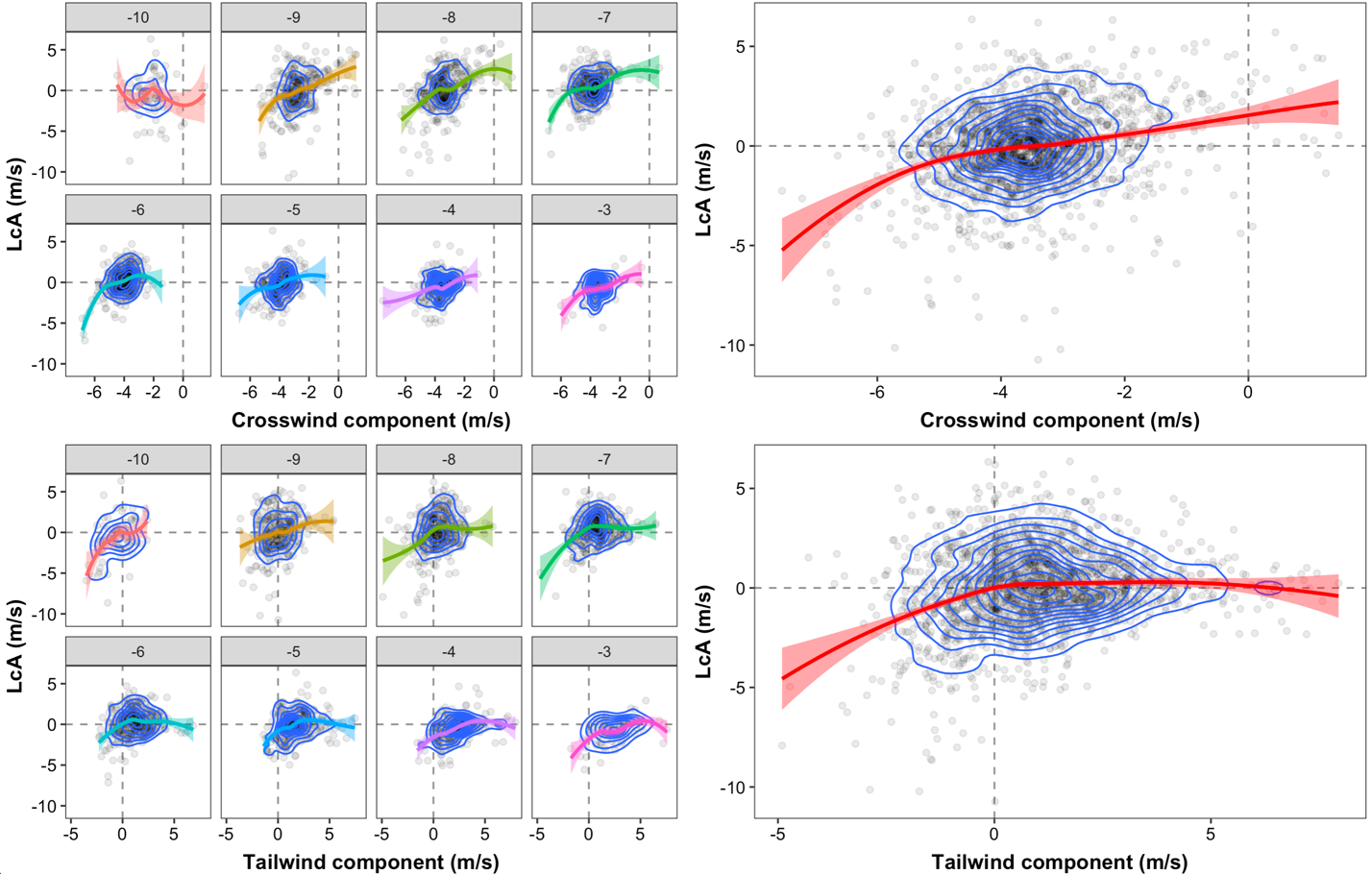


Fig. S5 – Relationships between lateral component of airspeed (LcA), Crosswind component (upper panels) and Tailwind component (lower panels) of the wind. Left panels represent these relationships according to “hour to sunset”, also highlighted by different colours of smoothed regression lines (loess function) and their 95% confidence intervals. A kernel density estimate was drawn to show the highest density of data points. Right panels represent the overall relationships between LcA and the two components of the wind.
